# WGCNA analysis revealing molecular mechanism that bio-organic fertilizer improves pear fruit quality by increasing sucrose accumulation and reducing citric acid metabolism

**DOI:** 10.3389/fpls.2022.1039671

**Published:** 2022-10-13

**Authors:** Zhonghua Wang, Han Yang, Yanwei Ma, Gaofei Jiang, Xinlan Mei, Xiaogang Li, Qingsong Yang, Jialiang Kan, Yangchun Xu, Tianjie Yang, Jing Lin, Caixia Dong

**Affiliations:** ^1^ Jiangsu Provincial Key Lab of Solid Organic Waste Utilization, Jiangsu Collaborative Innovation Center of Solid Organic Wastes, Educational Ministry Engineering Center of Resource-saving fertilizers, Nanjing Agricultural University, Nanjing, China; ^2^ Institute of Pomology, Jiangsu Academy of Agricultural Sciences/Jiangsu Key Laboratory for Horticultural Crop Genetic Improvement, Nanjing, China

**Keywords:** organic fertilizer, bio-organic fertilizer (BIO), sugar metabolism, TCA cycle, organic acid metabolism, fruit quality RNA-seq, WGCNA

## Abstract

It’s been long known that the application of organic fertilizer (OF) and bio-organic fertilizer (BF) which containing beneficial microorganisms to pear trees can both significantly improve fruit quality and yield. In order to reveal the mechanism of BF and OF regulating fruit growth and quality in pear, the effects of BF and OF on the photosynthetic characteristics and the accumulation of major sugars and organic acids of the pear fruit were quantified compared with chemical fertilizer (CF). Additionally, the molecular mechanisms regulating pear fruit development and quality were studied through transcriptome analysis. The three treatments were conducted based on the same amounts of nitrogen supply. The results showed that compared with CF, BF and OF treatments increased the fruit yield, and also significantly improved the photosynthesis efficiency in pear. BF and OF both significantly increased the sucrose content but significantly decreased the fructose and glucose content within the pear fruit. The amount of malic acid was significantly higher in OF treatment. Compared with CF and OF, BF significantly increased the sugar-acid ratio and thus improved the fruit quality. Transcriptome analysis and weighted correlation network analysis (WGCNA) revealed that the sugar metabolism of fruits applied with the BF was enhanced compared with those applied with CF or OF. More specifically, the expression of *SDH (Sorbitol dehydrogenase)* was higher in BF, which converts sorbitol into fructose. For both of the OF and BF, the transcript abundance of sugar transporter genes was significantly increased, such as *SOT (Sorbitol transporter)*, *SUT14 (Sugar transport 14)*, *UDP-GLUT4 (UDP-glucose transporter 4)*, *UDP-SUT (UDP-sugar transporter), SUC4 (Sucrose transport 4), SUT7 (Sugar transporter 7), SWEET10 and SWEET15 (Bidirectional sugar transporter)*, which ensures sugar transportation. The genes involved in organic acid metabolism showed decreased transcripts abundance in both BF and OF treatments, such as *VAP (Vesicle-associated protein) and cyACO (Cytosolic aconitase)*, which reduce the conversion from succinate to citric acid, and decrease the conversion from citric acid to malic acid in the TCA cycle (Tricarboxylic Acid cycle) through *Pept6 (Oligopeptide transporter).* In conclusion, the application of BF and OF improved fruit quality by regulating the expression of sugar and organic acid metabolism-related genes and thus altering the sugar acid metabolism. Both BF and OF promote sucrose accumulation and citric acid degradation in fruits, which may be an important reason for improving pear fruit quality. The possible mechanism of bio-organic fertilizer to improve fruit quality was discussed.

## Highlights

1. Compared with chemical fertilizer (CF), bio-organic (BF) and organic (OF) fertilizer can significantly promote sucrose accumulation and citric acid degradation in fruits, which may be an important reason for improving pear fruit quality.
2. Bio-organic fertilizer is more efficient for improving fruit yield than organic fertilizer, and less bio-organic fertilizer is required than organic fertilizer in promoting fruit growth.
3. Through metabolic and transcriptome profiling, 27 sugar/organic acid metabolism-related genes regulating pear fruit development were identified, including those involved in glycolysis and gluconeogenesis, TCA cycle, galactose metabolism, fructose and mannose metabolism, starch and sucrose metabolism pathways.

## Introduction

Sugar is the key component determining fruit quality and provides the fundamental materials for synthesizing fruit pigments, vitamins, amino acids and other quality and flavor substances (Cirilli et al., 2016; Dai et al., 2016). Sucrose, glucose, fructose and sorbitol are the main soluble sugars in pear fruit, which are also the most important indicators to measure the flavor quality of pear fruit (Moriguchi et al., 1992). Among them, the ratio of sucrose, fructose and glucose is an important factor to determine the sweetness of fruit (Colaric et al., 2005). Soluble sugar content and composition vary among different fruit developmental stages. Among different pear cultivars, the contents of sucrose and sorbitol vary more greatly, while the contents of glucose and fructose are relatively stable (Yao et al., 2010). In addition, the composition and content of organic acids are important factors to affect the quality and flavor of fruits. The main organic acids in most fruits include citric acid, malic acid, tartaric acid, and quinic acid. The types and contents of these organic acids are affected by the characteristics of different cultivars, production environments, and crop management (Liu et al., 2011). The composition and content of organic acids in fruit vary among different pear cultivars. The most abundant organic acid in the fruits of white pears, sand pears, *Pyrus ussuriensis* Maxim and *Pyrus sinkiangensis* Yü is malic acid, while the most abundant organic acid in most European pears is citric acid (Gao et al., 2004; Yao et al., 2010). Organic acids constantly change during development and are substances for glycolysis and the TCA cycle. In addition, organic acids are also involved in gluconeogenesis and play an important role during fruit ripening (Sha et al., 2011). The understanding of the effects of fertilizer on sugar and organic acid metabolism in fruit is crucial for controlling fruit quality.

By increasing soil organic substances and optimizing the rhizosphere environment, organic fertilizer enhances the nutrient absorption by roots and the accumulation of plant photosynthetic products, ultimately increasing crop yield and improving harvest quality (Morrissey et al., 2004). The increase of organic substances in the soil is beneficial for regulating fruit tree growth, and improving fruit yield and quality (Hogue et al., 2010; Choi et al., 2011; Song et al., 2012; Sas et al., 2014). Bio-organic fertilizer is a new type of organic fertilizer prepared by secondary solid-state fermentation of organic materials and specific beneficial microbial strains. Due to the different functional components added in, such as antibiotics, PGPR, and growth hormones, bio-organic fertilizer has different functions (Sathyapriya et al., 2012; Pieterse et al., 2014). Wang et al. (2017) studied the effects of bio-organic fertilizer on apples for 7 years and found out the following results: Compared with the control group (no fertilizer) and chemical fertilizer group, the soluble sugar content in fruits with bio-organic fertilizer was higher by 13.2%, and 6.3%, respectively. The reducing sugar content was higher in bio-organic fertilized apples by 53.9% and 5.9%, respectively. The Vitamin C content was increased by 6.1% and 3.8% respectively. Naher et al. (2021) found out that the application of bio-organic fertilizer containing *Bacillus cereus*, *Bacillus pumilus* and *Paenibacillus* spp. can promote the dissolution of phosphorus in soil and increase the rice yield by 65%. Kang et al. (2021) found out that bio-organic fertilizer can reduce the incidence of early leaf abscission in the pear and increase the yield of pear fruit in red soil by alleviating rhizosphere acidification, improving soil fertility, promoting lateral root development, and building a healthy rhizosphere microflora. The effect of fertilization on improving fruit quality has been widely studied, but the molecular mechanism regulating fruit sugar and organic acid metabolism in response to organic fertilization is still unclear.

Soil fertility is critical to the growth of pear trees. The application of organic or bio-organic fertilizer is an efficient way to improve soil fertility, which ensures balanced leaf nutrient content and higher fruit tree productivity (Xu et al., 2015). Organic and bio-organic fertilizer not only increases soil organic components but also promotes root development, which results in higher uptake efficiency of micronutrients in soil, such as Ca, Fe, and Zn. The application of organic or bio-organic fertilizer can significantly increase plant leaf area, photosynthetic rate, transpiration rate, and accumulation of K, Ca, Mg, and Fe in the leaf, thereby enhancing photosynthetic productivity and the growth of the aboveground parts (Agegnehu et al., 2016). Photosynthetic productivity has a restrictive effect on the growth, quality and yield of fruit trees. Wang et al. (2010) suggested that the level of *PAR* (*photosynthetically active radiation value*) and *Pn* (*net photosynthetic rate*) has significant effects on the yield and quality of fruits through regression analysis. It has been well accepted that the application of organic fertilizer plays an important role in fruit quality improvement. The mechanisms of fruit quality regulation by organic fertilizer are not clear so far.

With the improvement of technology and the update of bioinformatic databases, transcriptomics is playing an increasingly important role in fruit tree research (Wang et al., 2017; Xu et al., 2019). Through the combination of High-Performance Liquid Chromatography (HPLC) and quantitative real-time PCR (qRT-PCR) techniques, several genes regulating the accumulation of starch, organic acids, and soluble sugars in fruits have been identified, including *INV* (*Invertase*), *SS* (*Sucrose Synthetase*) and *SPS* (*Sucrose Phosphate Synthase*) (Zhang et al., 2010; Li et al., 2012). Lin et al. (2015) identified two up-regulated transporter genes possibly involved in the extracellular transport of citric acid during fruit development of ‘*Citrus reticulata*’ orange. The degradation of citric acid occurred mainly through the glutamine pathway, catalyzed by CitAco3-CitGS2-CitGDU1. Weighted gene co-expression network analysis (WGCNA) is an efficient method to describe the correlation patterns between phenotypic traits and genes in different samples. It can be used to identify clusters of genes with highly coordinated changes. It is currently an effective method to screen the associated biomarker genes between gene sets and phenotypic traits. Umer et al. (2020) used transcriptome profiling to study the co-expression patterns of gene networks related to sugar and organic acid metabolism in watermelon; three gene networks/modules and 2443 genes highly related to sugar and organic acid were identified, and 7 genes involved in sugar and organic acid metabolism were eventually mined through the WGCNA analysis. Although transcriptome technology has shown great advantages in the discovery of key regulatory genes and the analysis of metabolic pathways, the functions of genes are still unclear. In this study, RNA-seq sequencing technology was used to analyze the differences in fruit transcriptomes under different fertilization treatments, and WGCNA was used to analyze the relationship between the co-expressed gene modules associated with the physiological traits related to sugar and organic acid in fruit. The response of sugar and organic acid metabolites to organic and bio-organic fertilizers was also studied. The molecular characteristics of organic and bio-organic fertilizers regulating the main quality components of sugar and organic acid in fruits were revealed in this study, providing a theoretical basis for optimizing pear nutrient management through the combined application of chemical and organic fertilizers.

## Methods

### Experiment site overview

The experiment was performed at Lishui Plant Science Base of Jiangsu Academy of Agricultural Sciences (N31°36’59’’-E119°10’38’’), which locates in the transition zone from the north subtropics to the middle subtropics. There are four distinct seasons, mild and humid climate, plenty of rain, enough light, and a long frost-free period. The annual average temperature is 15.4°C, the annual sunlight duration is 2240 hours, the frost-free period is 237 days, and the average annual rainfall is 1087.4 mm.

### Experiment design

The new cultivar ‘Chuxialv’ of sand pear (*Pyrus pyrifolia*) was used in this study, which was planted from October 2018 to July 2020. The rootstock was *Pyrus betulifolia*, the pollinating tree was ‘Cuiguan’. ‘Chuxialv’ were planted on a Y-shaped trellis with a north-south direction. There are 50 trees planted per hectare, with a 2m × 5m distance between every two trees. The number of fruits on each tree was controlled at 150 with target yield of 3000kg/667m^2^. Grass grew naturally between the rows, and the tree’s architecture looks similar to each other. According to the equal nitrogen amount principle and the nitrogen content, three fertilization treatments were set up: chemical fertilizer (CF, control) (30 kg N/667m^2^, 15 kg P_2_O_5_/667m^2^, 35 kg K_2_O/667m^2^), ordinary organic fertilizer (OF) (1875 kg/667m^2^), and bio-organic fertilizer (BF) (1200 kg/667m^2^). Since the potassium provided in these two treatments was insufficient, it was supplemented with the chemical fertilizer K_2_SO_4_. Each treatment had 5 repeats, and each repeat was one tree. From October 2018 to October 2019, on both sides of the trees, 70 cm away from the trunk (near the drip line on the outer edge of the crown), trenches were opened respectively according to the size of 70 × 50 × 30 cm. The fertilizer and soil were fully mixed and backfilled, and other tree management was carried out as usual. Ordinary organic fertilizer (OF): organic matter ≥ 51.44%, the amount of N was 2.01%, P_2_O_5_ was 2.15%, K_2_O was 0.82%, water content 19.15%, pH 7.8. Bio-organic fertilizer (BF): *Bacillus amyloliquefaciens* which was effective strain ≥ 1×10^8^ CFU/g, the effective strain was *Bacillus amyloliquefaciens* SQR9; organic matter ≥52.93%, the amount of N was 3.12%, P_2_O_5_ was 5.44%, K_2_O was 0.78%, water content 17.02%, pH 5.8. Both organic and bio-organic fertilizers were produced by Jiangyin Lianye Biotechnology Co., Ltd. When the fruits were ripe (July 25th), 10 fruits were randomly picked from the middle part of the eastern and western sides of each tree, then the fruits were weighed with a balance and transported back to the laboratory with a loading box for further analysis. Following is nutritional amounts of different fertilization treatments:

1. CF, chemical fertilizer: during the young fruit stage (from late April to early May), the following fertilization was applied: topdressing 100 kg/667m^2^ of 15-15-15 compound fertilizer, 108.5 kg/667m^2^ urea (46% N), during fruit development (from late May to early June), topdressing 33.5kg/667m^2^ of potassium sulfate was applied.
2. OF, organic fertilizer: Base fertilization was applied 1875 kg/667m^2^ of ordinary organic fertilizer and 13 kg/667m^2^ of potassium sulfate, during fruit development, topdressing 19.5 kg/667m^2^ of potassium sulfate was applied.
3. BF, bio-organic fertilizer. Base fertilization was applied 1200 kg/667m^2^ of compound microbial fertilizer and 17 kg/667m^2^ of potassium sulfate, during fruit development, topdressing 25.5 kg/667m^2^ of potassium sulfate was applied.

### Vertical and horizontal diameter and hardness analysis

Vernier calipers were used to measure the vertical and horizontal diameters of fruits. The fruit hardness (skin hardness) was measured with a TA. XT. Plus (SMS, UK) texture analyzer on the middle of both sides of fruits, with a probe diameter of 8 mm, a test depth of 5 mm, and a penetration rate of 1 mm/s. The hardness was taken on two sites on each fruit, and the average value was used as the skin hardness of each fruit.

### Determination of mineral nutrient content

The quantification of mineral content in fruits was determined using the method of Shen et al (2018) with modifications. In 2020, fruit samples were weighed and boiled after harvesting. The mineral contents were measured by inductively coupled plasma atomic emission spectroscopy (ICP-AES). The mineral quantified in this study include nitrogen (N), phosphorus (P), potassium (K), calcium (Ca), boron (Ca), manganese (Mn), magnesium (Mg) and molybdenum (Mo).

### Determination of diurnal variation of photosynthesis and light response curve

After two years of fertilization treatments, the photosynthetic index was determined on a sunny day using a Li-6400 portable photosynthesis instrument (LI-COR, USA). The measurement was carried out once every 2 hours, from 7 a.m. to 5 p.m. The indicators measured include net photosynthetic rate (*P_n_*, μmol·m^-2^·s^-1^), transpiration rate (*T*_r_, mmol·m^-2^·s^-1^), intercellular CO_2_ concentration (*C_i_*, *μ*mol·mol^-1^), stomatal conductance (*G_s_*, mol·m^-2^·s^-1^). The daily integral value of net photosynthetic rate (Diurnal integral value of *P_n_*, DIV of *P_n_*) was calculated by Auto CAD software according to the area enclosed by the diurnal variation curve (Zhuang et al., 2006).

Light Response Curve: The experiment was carried out between 9 a.m. and 12 a.m. on a sunny day in mid-June 2021 using a Li-6400 portable photosynthesis instrument. For each treatment, 3 trees were selected, and 5 sun-faced leaves in the middle from both sides of trellises were randomly picked. The photosynthetic-induced light intensity before the measurement was 800*μ*mol·m^-2^·s^-1^. The gas circuit was open, and the flow setting value was 500 *μ*mol·s^-11^, and the CO_2_ concentration was 500 *μ*mol·s^-^. The photosynthetically active radiation value (*PAR*) of red and blue artificial light source (Li-6400-02B LED) was set at 2000, 1800, 1600, 1400, 1200, 1000, 800, 600, 400, 200, 150, 100, 50, 0 (*μ*mol·m^-2^·s^-1^). That is, the *P_n_-PAR* curve was measured in descending PAR order from 2000 *μ*mol·m^-2^·s^-1^ to 0 *μ*mol·m^-2^·s^-1^, and the readings were taken every 120s under each light intensity. *Pn-PAR* curve was analyzed by Photosynthesis Workbench analysis software and corrected by right-angle hyperbola correction model, as shown in formula (1)


(1) $$P_{n}(I)=\alpha \times \frac{1-\beta I}{1+\gamma I}\times I-R_{d}$$

In the formula, *α* is the initial quantum slope, *I* is the photosynthetically active radiation, *R_d_* is the dark respiration rate, *β* is the correction coefficient, and *γ* is the coefficient unrelated to the photosynthetically active radiation.

Linear regression was performed on the light response curve under weak light conditions (*I* ≤ 200 *μ*mol·m^-2^·s^-1^), and the obtained formular were used to calculate the apparent quantum efficiency (*AQY*), light compensation point (*LCP*), dark respiration rate (*R_d_*), the regression equation is as in formula (2)


(2) Pn=−Rd+AQY×I

### GC-MS analysis and determination of fruit carbohydrates

Fruit samples were collected for measurement when the fruits were ripe in 2020. Referring to Shen et al. (2018), the samples were freeze-dried in the vacuum and ground to fine powders with a grinder (MM 400, Retsch, 30 Hz, 1.5 min); 20 mg of tissue powder was extracted in 500 μL of extraction solution containing methanol, isopropanol and water (3:3:2 V/V/V). After vortexing for 3 minutes and centrifuged for 40 min at 14,000 for 3 minutes at 4°C, 100 μL of the supernatant was aspirated, 20μL of internal standard was added. The solvent was evaporated by drying under the nitrogen stream. 100 μL of methoxyammonium chloride in pyridine (15 mg/mL) were added and heated to 27°C for 2 hours. Then 100 μL of BSTFA was added, and the derivatization solution was obtained after incubation at 37°C for 30 min. After dilution with Hexyl hydride, it was stored in a brown injection vial for GC-MS analysis. Data processing was performed by using Agilent MassHunter qualitative and quantitative software.

### HPLC analysis and determination of fruit organic acids

Fruit samples were collected for metabolic profiling when the fruits were ripe in 2020. Referring to Shen et al. (2018), all samples were ground into fine powders in liquid nitrogen. After mixing, 5g of samples were weighed into a test tube, 10 mL of extraction solution (aqueous hydrochloric acid solution with pH 1 was added, and ultrasonic extraction was performed for 30 min. The supernatant was collected after centrifugation, the residue was added to 10 mL of extraction solution again, and ultrasonic extraction was performed for 30 min. The supernatant was collected after centrifugation and combined with the supernatant obtained previously, and 25 mL of the supernatant was taken and filtered with a syringe filter. The sample is ready for HPLC. Agilent 1100 high-performance liquid chromatograph equipped with Xtimate XB-C18 column (250mm*4.6mm, 5μm) was used for HPLC in this study (Agilent, USA). The condition of HPLC was set up as follows: the mobile phase: 0.01 moI/L of potassium dihydrogen phosphate solvent; G4212-60008 diode Array detector, detection wavelength: 215nm, the control flow rate: 0.8ml/min, column temperature: 40°C.

### RNA extraction, RNA-seq, and differential expression analysis

Every RNA sample was derived from five independent fruits, immediately frozen in liquid N2, and stored at −80 °C for further RNA-Seq assays. The total RNA from pear flesh was extracted using “RNAprep Pure Polysaccharide and Polyphenol Plant Total RNA Extraction Kit” (Tiangen Biochemical Technology Co., Ltd., DP441) and was completed according to its instructions. The transcriptome library was constructed using the “NEBNext1 Ultra™ RNA Library Prep Kit for Illumina” (NEB Corporation, USA) at Metware Biotechnology Co., Ltd. (Wuhan, China).

The original data obtained by high-throughput sequencing were converted into sequence data by CASAVA base calling. Each sample generated at least 6 gigabytes of data. For further analysis, the reads from the raw sequencing data were filtered, adaptors were removed, and low-quality (< Q30) reads trimmed. Then, the processed reads were mapped to the reference genome ‘Dangshansuli’ (*P. bretschneideri* Rehd.) (http://peargenome.njau.edu.cn/default.asp?d=4&m=2) with Tophat2 with the following parameters: segment length, 25, and segment mismatches, 2. Forthe remaining parameters, the default settings were used.

The uniformity, insert length, and saturation of the sequencing data were analyzed based on the alignment results. The number of reads aligned to each gene was calculated by HTSeq v0.6.1 software, and the Fragments Per Kilobase Million (FRKM) value was calculated by the following formula to measure the gene expression level: differential expression analysis between sample groups was performed by DEGSeq2. The adjusted p-value or false discovery rate (FDR) were calculated. The cutoff FDR of 0.05 was applied for all comparisons. A cutoff of 2 fold was applied for all comparisons.

### WGCNA analysis

Weighted gene co-expression network analysis was used to investigate the co-expression among genes associated with the sugar and organic acids content. The log2 transformation for the FPKM value was used for WGCNA analysis. The network constructions and module detections were performed with the following parameters: mergeCutHeight: 0.25, RsquaredCut: 0.85, TOMType: “signed”, and minModuleSize: 50. After all genes were categorized into different modules, the correlation between module eigen value and trait of interest was evaluated to identify modules related to sugar and organic acid content. The hub genes within the module associated with the trait of interest were identified based on the gene significance and module membership values (Zhang and Horvath, 2005).

### Statistics and analysis

SPSS 20.0 was used for statistical analysis. One-way analysis of variance (One-way ANOVA) combined with Duncan’s test was used to demonstrate the significance of the difference in means among multiple treatments.

## Results

### Effects of different fertilization treatments on fruit characters of pears at maturation period

**Figure 1:**
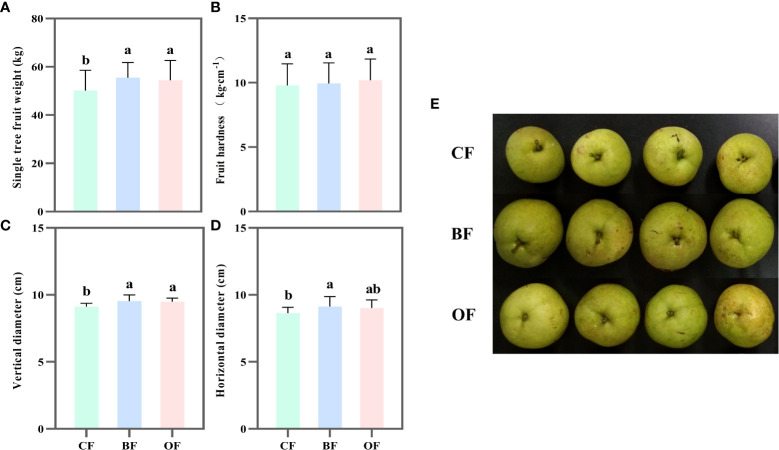
Effect of different fertilization treatments on fruit weight per tree **(A)**, fruit hardness **(B)**, fruit vertical **(C)**, horizontal **(D)** diameter and phenotype **(E)** of Pear. CF: chemical fertilizers application; BF: bio-organic fertilizers application; OF: organic fertilizers application. Values followed by different letters differ significantly (Duncan’s test, *P*< 0.05, n=5).

After two years of fertilization treatment, the yield of pear trees was estimated by weighing 150 fruits per tree. The average yield per pear tree treated with BF and OF was significantly higher than that of CF, by 10.6% and 8.4%, respectively (**Figure 1A**). There was no significant difference in fruit hardness under different fertilization treatments (**Figure 1B**). Under BF and OF treatments, the vertical diameter of fruit was increased by 5.8% and 4.4% (**Figure 1C**), respectively, and the horizontal diameter was increased by 4.5% and 4.3%, respectively, compared to that of CF (**Figure 1D**). That is to say, the application of both BF and OF increased the yield of pear free by increasing the fruit size (**Figure 1E**).

### Effects of different fertilization treatments on fruit element contents of pears at maturation period

**Figure 2:**
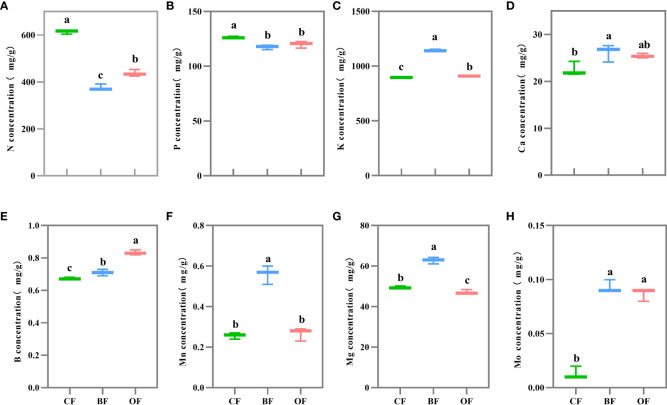
Effects of different fertilization treatments on the mineral contents of pear fruit. Values followed by different letters differ significantly **(A–H)**: Nitrogen concentration; Phosphorus concentration; Potassium concentration; Calcium concentration, Boron concentration; Manganese concentration; Magnesium concentration; Molybdenum concentration. (Duncan’s test, *P<* 0.05, *n = 5*). CF, OF and BF denote for chemical fertilizer, organic fertilizer and bio-organic fertilizer.

The mineral contents in pear fruit were changed significantly under different fertilization treatments (**Figure 2**). The N (nitrogen) content of fruit under CF treatment was significantly higher than that of BF (63.2%) and OF (40.3%) (**Figure 2A**), and the accumulation of P (phosphorus) under CF treatment was higher than that of BF (7.6%) and OF (5.3%) (**Figure 2B**). However, the accumulation of K (potassium) in pear fruit under BF treatment was significantly higher than that of CF (27.8%) and OF (26%) (**Figure 2C**), and the content of Ca (calcium), Mg (magnesium) and Mn (manganese) in fruit under BF treatment was higher compared with other treatments (**Figure 2D**). The content of B (boron) in OF treatment was higher than that in BF and CF (**Figure 2E**). The content of Mo (molybdenum) in CF treatment was significantly lower than in other treatments (**Figure 2H**).

### Effects of different fertilization treatments on photosynthetic parameters of pear leaves at maturation period

**Figure 3:**
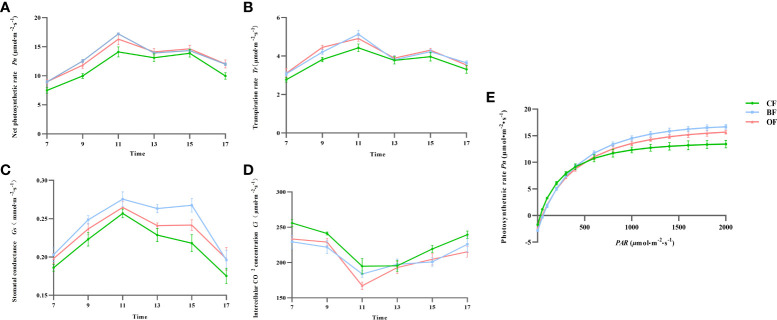
Fitting curves of *T_r_* **(A)**, *P_n_***(B)**, *G_s_***(C)**, *C_i_***(D)** from 7 a.m. to 5 p.m and light response **(E)** from 9 a.m. to 12 a.m. in pear leaves with different fertilization treatments. CF, OF and BF denote for chemical fertilizer, organic fertilizer and bio-organic fertilizer, respectively. *Pn*, photosynthetic rate; *Tr*, transpiration rate; *Ci*, intercellular CO_2_ concentration; *Gs*, stomatal conductance.

Improving the photosynthetic capacity of leaves is an important way to improve crop yield and fruit quality. At 107 days after blooming, the value of *P_n_* in pear displayed a “double-peak” curve under all three fertilization treatments, demonstrating the obvious phenomenon of photosynthetic ‘lunch break’ (**Figure 3A**). There was no significant difference of *P_n_* between OF and BF treatments, but the *P_n_* values of OF and BF were significantly higher than that of CF treatments (*P*<0.05). Compared with CF treatment, BF and OF treatments significantly promoted the net photosynthetic rate of pear leaves during fruit development. In the same period, the diurnal variation curve of *T_r_* and the diurnal variation of *G_s_* in pear leaves were similar for different fertilization treatments (**Figures 3** **B, C**). Compared with CF treatment, pear leaves in BF and OF treatments had higher stomatal conductance during fruit development (**Figure 3D**).

The light response curve reflected the adaptability of plants to external light and environmental conditions. Before reaching the light saturation point, the net photosynthetic rate of pear leaves in different fertilization treatments showed a trend of increasing first and then tending to be stable (**Figure 3E**). When *PAR* was below 800*µ*mol·m^-2^·s^-1^, there was no significant difference in the variation of the *Pn-PAR* curve among the treatments; when *PAR* was above 800*µ*mol·m^-2^·s^-1^, there was a significant difference in the variation of the *Pn-PAR* curve among the treatments, that is, the changes of the *Pn-PAR* curve in the BF and OF treatments were significantly higher than those in the CF treatment (*P<* 0.05). Compared with CF treatment, BF and OF treatments can significantly increase the photosynthetic rate of pear leaves.

### Effects of different fertilization treatments on sugar and organic acids of pear fruits at maturation period

**Figure 4:**
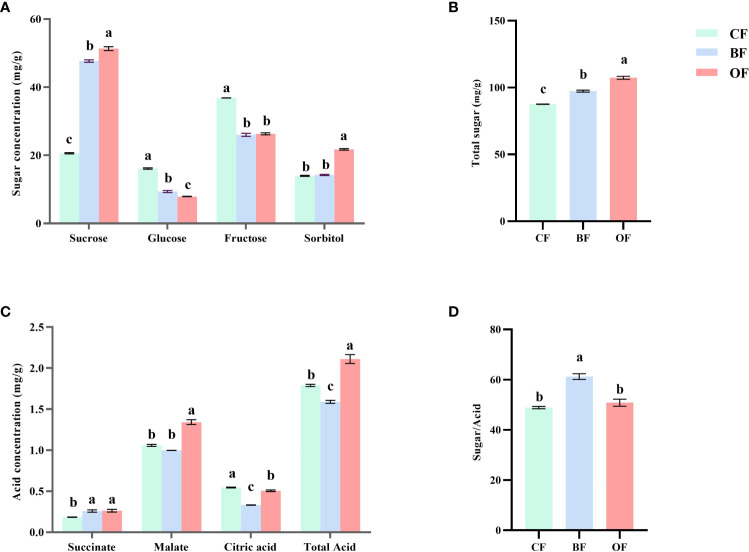
Effect of different fertilizer treatments on soluble sugar **(A)**, total sugar **(B)**, organic acids **(C)** and sugar/acid ratio **(D)** of pear fruits at the mature Stage. CF, OF and BF denote for chemical fertilizer, organic fertilizer and bio-organic fertilizer, respectively. Values followed by different letters differ significantly (Duncan’s test, *P*< 0.05, *n=5*).

As shown in **Figure 4**, the sugar & organic acid content and sugar-acid ratio in the fruit changed significantly under different fertilization treatments. Compared with CF treatment, BF and OF treatments significantly (*P*< 0.05) increased the total content of fructose, sorbitol, glucose, and sucrose, and their total sugar content increased by 11.2% and 22.6% (**Figure 4B**), respectively. In terms of specific sugar components, BF and OF treatments increased the contents of sucrose and sorbitol, of which sucrose content was increased by 131.5% and 149.2% compared with CF, respectively, and sorbitol content was increased by 21.7% and 2.1%, respectively. As for the hexose content, the fructose and glucose contents under BF and OF treatments were significantly decreased compared to CF, in which glucose content was decreased by 42% and 50.8%, respectively, and fructose content was decreased by 29.4% and 28.5%, respectively (**Figure 4A**).

As shown in **Figure 4C**, the most abundant organic acid in the fruit of ‘Chuxialv’ pear is malic acid, followed by citric acid and succinic acid. Compared with CF treatment, the content of malic acid in ripe fruit was significantly increased by 26.8% under OF treatment, and there was no significant difference between BF treatment and CF treatment. Succinic acid content was increased by 40.5% and 42.7%, respectively, under BF and OF treatments. The citric acid content under BF and OF treatments was decreased by 64% and 8.1%, respectively. Compared with CF treatment, the total acid content was the lowest under BF treatment and highest under OF treatment. The sugar-acid ratio was an important indicator of fruit quality, and the sugar-acid ratio under BF treatment was significantly higher than that under CF and OF treatments, suggesting that bio-organic fertilizer is the most efficient fertilizer for increasing the sugar-acid ratio of the pear fruit, which is of great significance for improving the fruit quality (**Figure 4D**).

### Identification of DEGs co-expression modules by WGCNA

**Figure 5:**
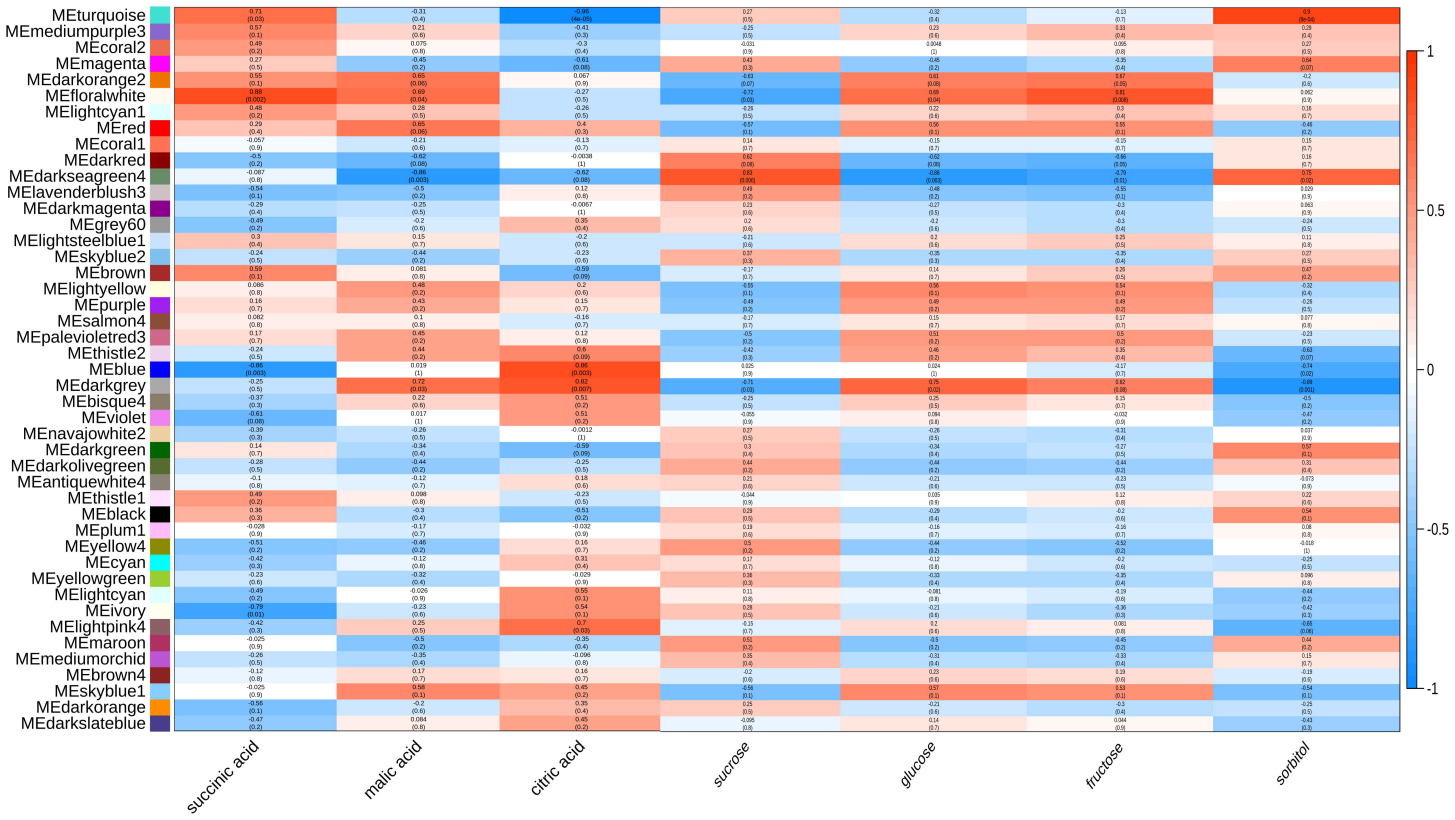
Weighted gene co-expression network analysis among genes associated with the sugar and organic acids content. Module-trait correlations and corresponding P values were presented. Each column represents a physiological trait, and each row represents a genetic module. The color scale shows module trait correlations from -1 (blue) to 1 (red).

WGCNA was performed for all genes with a FPKM value greater to 5, resulting in the identification of 45 co-expression modules as shown in **Figure 5**. The module-trait relationship was investigated to explore modules associated with traits of interest including the content of sugar and organic acids. The modules were divided according to the clustering relationship between genes, and then the modules with similar expression patterns were merged according to the similarity of module eigengenes.

**Figure 6:**
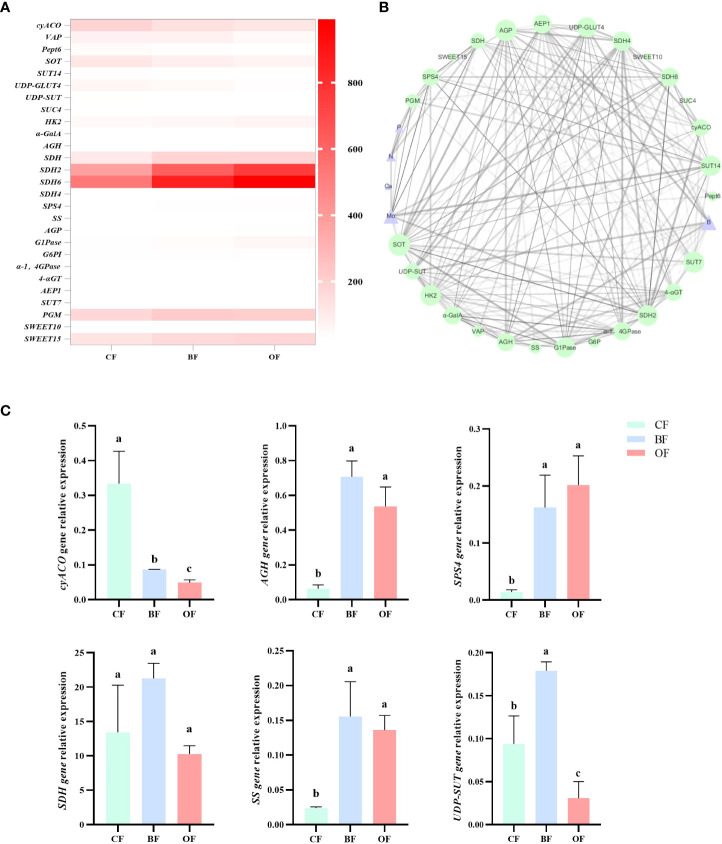
**(A)** Heatmap of candidate hub genes for regulation of sugar and organic acid metabolism in the MEBlue, MEturquoise and MEfloralwhite modules. CF: chemical fertilizers application; BF: chemical fertilizers + bio-organic fertilizers application; OF: chemical fertilizers + organic fertilizers application. The FPKM values of CF, BF and OF from white (5) to red (1000). **(B)** Construction of regulatory networks of sugar and organic acid metabolism gene (green) in the MEBlue, MEturquoise and MEfloralwhite module, which positively correlated with nutrient contents (purple) (r^2^>0.7). Hub genes (key candidates) within each network are highlighted in size due to the highest weight within the module and coded for gene descriptors based on annotations, the strength of correlation between hub genes by weights of lines. **(C)** Quantitative RT-PCR data of a sub-set of genes differentially expressed in the fruits in response to fertilizer. The experiments were repeated three times Values followed by different letters differ significantly. The error bars represent mean ± SE (P>0.05, n = 3).

By calculating the correlation between the eigenvalues and traits of each module, it was found that the MEdarkgrey, MEdarkseagreen4, MEBlue, MEturquoise and MEfloralwhite modules were highly correlated with the contents of sugar and organic acids (*P*>0.7) (**Figure 5**). By analyzing the enrichment of sugar and organic acid metabolism-related genes, it was found that within MEturquoise module, there was the largest number of DEGs involved in sugar and organic acid metabolism, and there were 18 DEGs related to sugar and organic acid metabolism, which illustrates that MEturquoise module could be related to the sugar and organic acid metabolism. Through the analysis of gene expression, KEGG pathway, and DEG in these modules, 27 candidate hub genes were screened with the consistent change trend of sugar and organic acid content (**Table 1**; **Figure 5**). Through the heat map analysis of the expression number of these 27 candidate hub genes, it was found that there were 3 acid metabolism-related genes, 16 sugar metabolism-related genes, and 8 sugar transport-related genes, among which *SDH4 (Sorbitol dehydrogenase 4)* and *SDH6* had higher expression levels. The gene co-expression network formed by the above genes responded to the changes in sugar-acid traits in fruits under the regulation of bio-organic fertilizer and ordinary organic fertilizer application (**Figure 6**).

**Table 1:** Candidate hub genes for regulation of sugar and organic acid metabolism in the MEBlue, MEturquoise and MEfloralwhite modules.

Modules	Gene	Gene ID	Description
MEblue	cyACO	Chr9.g46434	Cytosolic aconitase
MEblue	VAP	Chr3.g18389	Ve.sicle-associated protein 4-1
MEblue	Pept6	Chr7.g31646	Oligopeptide transporter 6
MEblue	SOT	Chr10.g16531	Sorbitol transporter
MEblue	SUT14	Chr15.g03127	Sugar transport 14
MEblue	UDP-GLUT4	Chr2.g42980	UDP-glucose transporter 4
MEblue	UDP-SUT	Chr15.g03811	UDP-sugar transporter
MEblue	SUC4	Chr8.g55897	Sucrose transport 4
MEturquoise	HK2	Chr15.g04826	Hexokinase-2
MEturquoise	a-GalA	Chr16.g30552	a-Galactosidase
MEturquoise	AGH	Chr10.g14669	a-Glucosidase
MEturquoise	SDH	Chrl.g56757	Sorbitol dehydrogenase
MEturquoise	SDH2	Chrl.g56758	Sorbitol dehydrogenase
MEturquoise	SDH6	Chr7.g31957	Sorbitol dehydrogenase
MEturquoise	SDH4	Chr7.g31956	Sorbitol dehydrogenase
MEturquoise	SPS4	Chr10.g17334	Sucrose-phosphate synthase 4
MEturquoise	ss	Chr15.g02866	Sucrose synthase
MEturquoise	AGP	Chr17.g25838	Glucose-1-phosphate adenylyltransferase
MEturquoise	GJPase	Chr5.g08695	Glucose-1-phosphate adenylyltransferase
MEturquoise	G6PI	Chr8.g53896	Glucose-6-phosphate isomerase
MEturquoise	a-1 , 4GPase	Chr17.g25148	a-1,4 Glucan phosphorylase
MEturquoise	4-aGT	Chr15.g01502	4-a-Giucanotransferase
MEturquoise	AEPJ	Chr17.g25022	Aldose 1-epimerase
MEturquoise	SUT7	Chr11.gl3237	Sugar transporter 7
MEfloralwhite	PGM	Chr16.g31272	Phosphoglucomutase
MEfloralwhite	SWEETJO	Chr6.g51704	Bidirectional sugar transporter
MEfloralwhite	SWEET15	Chr16.g30311	Bidirectional sugar transporter

CF: chemical fertilizers application; BF: chemical fertilizers + bio-organic fertilizers application; OF: chemical fertilizers + organic fertilizers application. The FPKM values of CF, BF and OF from blue (low) to red (high).

To understand the regulatory network between fruit mineral content and sugar and organic acid metabolism genes, 27 sugar acid metabolism-related genes were screened among MEBlue, MEturquoise and MEfloralwhite modules (**Table 1**; **Figures 5**, **6**), and the relationship between the mineral content and all genes are shown by using a Pearson correlation coefficient threshold greater than 0.8 (**Figure 6**). The visualization results of Cytoscape showed that there were 36 nodes connected with 1096 edges in the regulatory network of mineral content and sugar and organic acid metabolism (**Figure 6**). Based on the margin cutoff of 10, we found that the minerals B and Mo had higher correlations with sugar and organic acid-related gene expression.

### Effects of different fertilization treatments on the transcriptome of pear fruit sugar and organic acid by KEGG analysis

**Figure 7:**
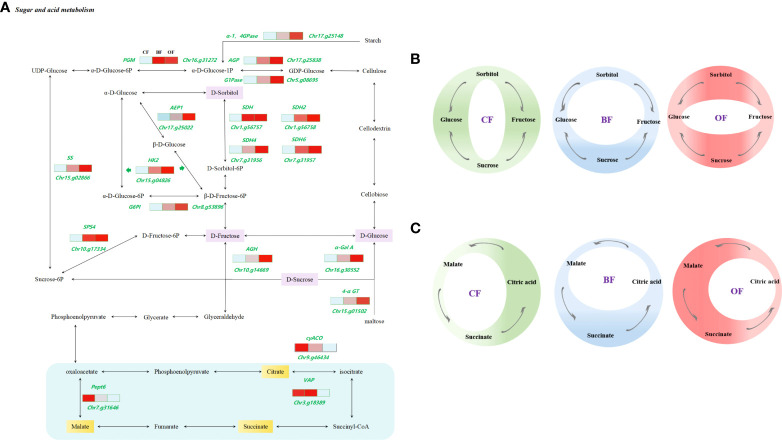
Relationship between KEGG pathway with sugar/organic acid metabolism. **(A)** Expression profiles of differentially expressed genes (DEGs) involved in sugar and organic acid metabolism in pear fruit from KEGG pathways. Enzyme names are shown along with their expression patterns at different fertilization treatments. Grids represent the expression patterns of genes shown as FPKM values: CF, BF, OF. **(B, C)** Effect of different fertilizers on sugar and organic acid metabolism transformations in fruits. The circle area represents the extent of sugar accumulation.

Sorbitol and sucrose are the major sugars in pear. The accumulation of sugars in pear fruits is mainly regulated by the catabolism and anabolism of sorbitol and sucrose. The dominant sugars in pear fruit under the CF treatment were glucose and fructose, and the sorbitol and sucrose content were relatively low (**Figure 4**). The accumulation of sucrose was significantly increased by BF and OF treatments, and sorbitol content was also increased under OF treatment (**Figure 7B**). The application of OF and BF promoted the accumulation of total sugar content in fruits, mainly through sucrose content, implying significant differences in sugar metabolism between organic and chemical fertilizer treatments. The three major organic acids, including malic acid, citric acid, and succinic acid, are important metabolites of the TCA in plants. Under BF treatment, citric acid and total acid content were reduced by increased succinic acid content. Under OF treatment, the total acid content was increased by promoting the accumulation of succinic acid and malic acid (**Figure 7C**). Among all the annotated pathways in sugar and organic acid metabolism and transcriptional regulatory network analysis, we mainly focus on glycolysis and gluconeogenesis, TCA cycle, galactose metabolism, fructose, and mannose metabolism, starch and sucrose metabolism. There were significant differences in the expression of genes involved in sugar acid synthesis and catabolism among three different fertilization treatments (**Figure 7A**). The expression of sugar-metabolism-related genes in the KEGG pathway under OF and BF treatment was significantly higher than that under CF treatment, and the expression of acid metabolism-related genes was significantly lower than that under CF treatment, which is consistent with the conclusion in **Figure 4** that the application of two organic fertilizers significantly improved the sugar-acid ratio of fruits.

Carbohydrates produced by photosynthesis are mainly converted into hexoses by synthases or invertases. The application of OF and BF increased the expression level of *AGH* (*α-Glucosidase*) and *α-GalA* (*α-Galactosidase*) and promoted the conversion of sucrose into fructose and glucose. The expression level of *SDH* (*Sorbitol dehydrogenase, Sorbitol dehydrogenase* 2*, Sorbitol dehydrogenase* 4*, Sorbitol dehydrogenase* 6) was also increased under OF and BF application, which is responsible for converting sorbitol into fructose. Additionally, *AEP1* (*Aldose 1-epimerase*), *PGM* (*Phosphoglucomutase*), *AGP* (*Glucose-1-phosphate adenylyltransferase*) and *G1Pase* (*Glucose-1-phosphate adenylyltransferase*), *G6PI* (*Glucose-6-phosphate isomerase*), *HK2* (*Hexokinase* 2), *SS* (*Sucrose synthase*), and *SPS4* (*Sucrose-phosphate synthase* 4) regulate the transformation between hexose sugars, further promoting the accumulation of sugars. In addition, the decomposition of macromolecular polysaccharides during fruit ripening was an important way to increase the sugar content of fruit. BF and OF treatments could promote the conversion of starch and maltose to glucose by *α-1, 4GPase* (*α-1, 4 Glucan phosphorylase*) and *4-αGT* (*4-α-Glucanotransferase*). In addition, the transcript abundance of sugar transporter genes was significantly increased, such as *SOT (Sorbitol transporter)*, *SUT14 (Sugar transport* 14*)*, *UDP-GLUT4 (UDP-glucose transporter* 4*)*, *UDP-SUT (UDP-sugar transporter), SUC4 (Sucrose transport* 4*), SUT7 (Sugar transporter* 7*), SWEET10 and SWEET15 (Bidirectional sugar transporter)*, which ensures sugar transportation. Organic acid metabolisms play an important role on affecting fruit flavor. The expression of *VAP* (Vesicle-associated protein) and *cyACO* (*Cytosolic aconitase*) under BF and OF treatments was reduced, thus inhibiting the transformation of succinic acid to citric acid and improving the fruit flavor by reducing citric acid content. OF treatment could promote the conversion of citric acid to malic acid in the TCA cycle by reducing the expression level of *Pept6* (*Oligopeptide transporter*) (**Figure 7**; **Table 1**). In addition, quantitative PCR analyses further confirmed that the expression of hub genes (**Figure 6B**).

## Discussion

### Effects of organic fertilizer application on fruit quality and sugar and organic acid components

Previous studies showed that the excessive application of chemical fertilizer is one of the major reasons leading to environmental degradation and global warming. Continuous application of organic fertilizer or bio-organic fertilizer can improve soil fertility and significantly increase fruit tree yields (Mosa et al., 2015; Wang et al., 2017). Organic fertilizer and bio-organic fertilizer have been proven to be effective in promoting crop yields (Liu et al., 2021). Bio-organic fertilizer containing plant growth-promoting rhizosphere bacteria (PGPRs) can improve soil fertility and plant growth, thereby increasing crop yields (Liang et al., 2020). The results of this study showed that the application of BF and OF significantly increased the yield of pears compared to CF, confirming that both organic fertilizer and bio-organic fertilizer could increase the yield of mature pear trees. Interestingly, BF had greater potential for sustained yield increase than OF. Fruit hardness was one of the important indicators to measure the quality and maturity of pear fruit. It not only affected the taste of fresh fruit but also affected the quality in the period of post-harvest storage and shelf life. Fertilization and hormone treatment could both affect fruit firmness. Nutrients such as P, K and Ca were beneficial for maintaining fruit hardness. Although more nitrogen fertilization could promote the yield increase, fruit hardness tended to be decreased. The data in this study showed that there was no significant difference in fruit hardness among CF, OF, and BF treatments. Therefore, compared with CF treatment, OF and BF treatments increased the yield of pear fruit and improved the pear fruit quality. The fruit hardness was not affected by OF or BF (**Figure 1**).

Organic fertilizer promotes plant root growth by changing soil pH, increasing organic matters in soil, available nutrients, trace element content, soil porosity, and soil permeability (Kang et al., 2021). Bio-organic fertilizer is a new type of organic fertilizer prepared by secondary solid-state fermentation of organic materials and specific functional microbial strains. It has been widely used in apples (Wang et al., 2016), bananas (Shen et al., 2019), pepper trees (Yu et al., 2019), and kiwis (Liu et al., 2020). Many strains within the rhizosphere had beneficial effects on plant growth, resistance, and nutrient uptake by mineralizing organic matter (Tiwari et al., 2019). In our study, after two years of fertilization treatments, the mineral contents in the fruits changed significantly (**Figure 2**). N and P concentrations in the fruits under the CF treatment were significantly higher than those under the BF treatment and OF treatment, and the application of OF could significantly increase the photosynthesis capacity and amounts of K, Ca, Mg and Mn in the fruits. The amount of B and Mo was elevated as well. It is possible that the application of OF or BF increased the content of trace elements in the fruits and thus contributed to the improvement of fruit quality. Plant biomass and photosynthetic capacity were the major determinants of crop yield and fruit quality. Crop management could directly or indirectly improve crop photosynthetic capacity (Makino, 2011). Improving leaf photosynthetic capacity was an important way to achieve high yield and improve fruit quality (Murchie et al., 2009; Evans, 2013; Dann and Leister, 2017), so it could be inferred that the significant improvement of photosynthetic capacity under BF and OF treatments (**Figure 3**) was the premise of fruit carbohydrate accumulation and fruit quality improvement.

The sweetness was the main parameter that determined the sensory quality of fruit, and the intensity of sweetness is mainly determined by the composition and content of sugar. Different sugars were involved in different biological events. The increase in the ratio of sucrose/glucose content was the reason for promoting cell division (Gibson, 2005). In this study, compared with the CF treatment, the sucrose content under the BF and OF treatments was significantly increased (**Figure 4**), which may also be one of the important reasons for the increase in single fruit weight by promoting cell division. In citrus plants, sucrose synthesis shifted to decomposition at the final stage of maturation and glycolysis and tricarboxylic acid cycle were accelerated, and the flow of carbohydrates toward energy metabolism was enhanced (Lin et al., 2015). In this study, the total soluble sugar content under BF treatment and OF treatment was significantly higher than that under CF treatment (**Figure 4**), but the total sugar content in the fruits under BF treatment was lower than that under OF treatment, which might be related to the less amount of bio-organic fertilizer applied in this study. The amount of BF applied was only 64% of the amount of OF, and the organic matter content in BF was 28.44% of that of ordinary organic fertilizer. The experiment site had relatively sticky and heavy soil, and the application of more organic matter could have a more effect on the accumulation of the total sugar content in the fruits. Sugar and organic acid metabolism were closely linked to each other through the TCA cycle. Organic acid was the material basis of the TCA cycle and glycolysis, and played an important role during fruit ripening. The accumulation of organic acid was the result of its synthesis, degradation, utilization and regionalization (Li et al., 2021). In this study, the main organic acids in pear fruit were malic acid and citric acid. Compared with CF treatment, OF treatment significantly reduced citric acid content while it significantly increased malic acid content, resulting in a significant increase in total acid content. Under BF treatment, citric acid content was decreased significantly, and the amount of total organic acid was reduced (**Figure 4**). Although the total sugar content under BF treatment was not as high as that under OF treatment, the sugar-acid ratio was significantly higher than that under OF treatment (**Figure 4**), which significantly improved fruit flavor. This indicated that OF and BF may have different mechanisms in regulating fruit sugar and organic acid metabolism. At present, the effect of microorganisms on fruit quality is still unclear. Both OF and BF played an important role in regulating fruit quality, probably due to the complexity of the components contained in OF and BF, as well as the significantly different composition characteristics from CF. The effects of the two organic fertilizers on fruit quality in this study may be related to the role of functional microorganisms in bio-organic fertilizer, and may also be related to the large difference in the amount of organic matter brought by the two organic fertilizers, which needs further research.

### Transcriptome analysis reveals the molecular mechanisms regulating sugar and organic acid content in response to organic fertilization

With the rapid development of modern molecular biology and bioinformation, biological research has entered the era of big data in systematic biology. Transcriptomics technologies are the techniques used to study an organism’s transcriptome, the sum of all of its RNA transcripts. It is regulated by endogenous and exogenous factors at the same time. Transcriptomics is a bridge between the genome genetic information and the functional proteome. The transcriptome analysis of the quality of different pear cultivars showed that the pear cultivars with higher sugar content had higher *SDH* expression and ratios of sorbitol and fructose (Dai et al., 2015). Therefore, the transcript level of *SDH* is significantly associated with fruit flavor, which was verified in a comparison of 234 cultivated apple cultivars with 20 wild apple cultivars. The wild apples displayed a lower expression level of *SDH* compared with the cultivated apples (Fang et al., 2020). SDH was decomposed into fructose by SDH dehydrogenase, and SDH could promote sorbitol metabolism and regulate SS and SPS activities, and thus increasing the accumulation of sucrose in fruits, which was beneficial for improving fruit quality (Yu et al., 2021). Fruit development and maturation were complex biological processes regulated by genetic and environmental factors. The flavor characteristics of fruit were jointly determined by sugar and organic acid content. The accumulation of citric acid was also the main factor responsible for the sour flavor of pear fruits. ACO-encoded aconitase was the first step of citric acid catabolism, which played a major role in the acidity change of pineapple fruit before harvest (Saradhuldhat and Paull, 2007; Terol et al., 2010; Liu et al., 2011). The over expression of *VAPs* increased the plant biomass and carbohydrate products in poplar (Gandla, 2021; Singh et al., 2018). WGCNA revealed the sugar and organic acids related metabolism under different fertilization conditions. The results showed that both BF and OF treatments significantly increased the expression level of *SDH*, *SS*, and *SPS*, resulting in the accumulation of sucrose. OF treatment also significantly promoted the accumulation of sorbitol (**Figure 3**; **Figure 7B**). Citric acid content was significantly reduced under BF and OF treatments, consistent with the decreased expression of *VAP* and *cyACO*, that is to say, the transformation amount of succinate to citric acid was reduced (**Figure 7A**). It was consistent with the response of pear fruit to different organic fertilizer treatments at the physiological level.

The fruit flavor is a complicated trait and is determined by many factors. Sorbitol was the major type of sugar in *rosaceae* plants, and it was a transitional storage substance in fruit sugar metabolism. In the fruits, sorbitol was converted to glucose and fructose, which can be used for sucrose and starch synthesis (Li et al., 2018). In this study, it was found that the application of OF and BF improved the fruit quality by increasing the sugar-acid ratio, promoting the accumulation of sucrose and the decomposition of citric acid. The amount of fructose and glucose showed a decreasing trend under the two organic fertilizer treatments (**Figure 3**). The results implied different mechanisms for the accumulation of sugars under different fertilizer treatments. The organic and bio-organic fertilizer had the potential to improve fruit flavor. Two genes responsible for the conversion of sucrose into fructose and glucose, *AGH* (*α-Glucosidase*) and *α-GalA* (*α-Galactosidase*), displayed significantly increased transcript levels after the application of organic and bio-organic fertilizer. In the study of melon fruit quality, *G6PI* regulated the transformation between glucose and fructose and affected the quality and taste of melon (Lebeda and Paris, 2004). In this study, AEP1 and PGM acted as mutarotase proteins, catalyzed the transformation of glucose to other aldoses, and were involved in hexose metabolism. The expression level of *AEP1* and *PGM* was elevated after organic and bio-organic fertilizer treatment. HK acted as a glucose signaling molecule and a catalyst of glycolysis, and the HXK protein altered by HK was involved in phosphorylation, providing ATP and metabolites for plants, which was inseparable from the carbon cycle (Zhao et al., 2019). In this study, the expression level of *HK2* gene was significantly increased under the BF and OF application, which enhanced the transformation between glucose and fructose. To ensure the normal operation of sugar metabolism, the expressions level of *SOT, SUT14, UDP-GLUT4, UDP-SUT, and SUC4* sugar transporter proteins were significantly decreased under BF and OF treatment, and the expression level of *SUT7, SWEET10* and *SWEET15* was significantly increased, regulating the transport of sugar (**Figure 7A**). In summary, the application of organic and bio-organic fertilizer not only changed the accumulation of sugar in the fruits but also made the hexose metabolism in the fruits more active. Therefore, the change of sugar and organic acid content in the fruits was not just regulated by a few genes but by joint efforts of multiple metabolic pathways.

## Conclusions

After two consecutive years of fertilization treatments, it was found that compared with chemical fertilizer, both ordinary organic fertilizer and bio-organic fertilizer could significantly improve fruit quality and flavor. Compared with chemical fertilizer and ordinary organic fertilizer, the effect of bio-organic fertilizer on sugar and organic acid metabolism was moderate, but it had a significant effect on fruit quality improvement. Through the analysis of fruit transcriptome under different fertilizer treatments, 27 candidate genes of sugar and organic acid metabolism in pear fruit in response to organic fertilizer were screened, which laid a foundation for further research on sugar and organic acid metabolism in pear fruit. At the same time, this study also provided the theoretical and practical approach for reducing chemical fertilizer and increasing bio-organic fertilizer for pear trees. The specific regulatory mechanism of candidate genes still needs to be studied in the future, and the regulation of bio-organic fertilizers in fruit quality also needed further in-depth research.

## Data availability statement

The original contributions presented in the study are publicly available. This data can be found here: NCBI, PRJNA882780.

## Author contributions

ZW and CD conceived and designed the experiments; XL, QY and JK performed the experiments; TY, YM and XM analyzed the data; JL, GJ and YX contributed reagents/materials/analysis tools; ZW wrote the paper. HY interpreted data and edited the manuscript. All authors read and approved the final manuscript.

## Funding

This research was supported by the Science and Technology Support Program of Jiangsu Province (BE2019374); The earmarked fund for Jiangsu Agricultural Industry Technology System (JATS[2021]437), and the earmarked fund for CARS (CARS-28).

## Conflict of interest

The authors declare that the research was conducted in the absence of any commercial or financial relationships that could be construed as a potential conflict of interest.

## Publisher’s note

All claims expressed in this article are solely those of the authors and do not necessarily represent those of their affiliated organizations, or those of the publisher, the editors and the reviewers. Any product that may be evaluated in this article, or claim that may be made by its manufacturer, is not guaranteed or endorsed by the publisher.
